# Identifying New Positives and Linkage to HIV Medical Care — 23 Testing Site Types, United States, 2013

**Published:** 2015-06-26

**Authors:** Puja Seth, Guoshen Wang, Nicoline T. Collins, Lisa Belcher

**Affiliations:** 1 Division of HIV/AIDS Prevention, National Center for HIV/AIDS, Viral Hepatitis, STD, and TB Prevention, CDC

Among the estimated 1.2 million persons living with human immunodeficiency virus (HIV) infection in the United States, approximately 14% have not had their HIV diagnosed (1). Certain populations, such as African Americans/blacks (in this report referred to as blacks), men who have sex with men (MSM), and Hispanics/Latinos (in this report referred to as Hispanics), are disproportionately affected by HIV. In areas where HIV prevalence is ≥0.1%, CDC recommends routine HIV screening in health care settings for persons aged 13–64 years. Implementation of HIV screening as part of routine care can increase the number of HIV diagnoses, destigmatize HIV testing, and improve access to care for persons with new HIV infections (2). Additionally, targeted testing in non–health care settings might facilitate access to persons in at-risk populations (e.g., MSM, blacks, and Hispanics) who are unaware of their status and do not routinely seek care (3). CDC analyzed data for 23 testing site types submitted by 61 health departments and 151 CDC-funded community-based organizations to determine 1) the number of HIV tests* conducted, 2) the percentage of persons with new diagnoses of HIV infection (in this report referred to as new positives), and 3) the percentage of persons who were linked to HIV medical care within 90 days after receiving diagnoses at specific site types within health care and non–health care settings. The results indicated that, in health care settings, primary care and sexually transmitted disease (STD) clinics accounted for substantially more HIV tests than did other sites, and STD clinics identified more new positives. In non–health care settings, HIV counseling and testing sites accounted for the most tests and identified the highest number of new positives. Examining program data by site type shows which sites performed better in diagnosing new positives and informs decisions about program planning and allocation of CDC HIV testing resources among and within settings.

In 2013, CDC funded 61 health departments† and 151 community-based organizations to provide HIV testing and HIV prevention services in the United States. Data on CDC-funded HIV testing and other HIV prevention activities are collected locally. Required data are submitted via a secure CDC-supported web-based data system. CDC and grantees use these data for monitoring and evaluation of HIV testing and service delivery.

Valid HIV tests are records in which data on test technology (conventional, rapid, nucleic acid amplification testing, or other) or test result (positive, negative, indeterminate, or invalid) or both are reported. Persons who test positive for HIV and report no prior positive test results are categorized as new positives. Linkage to HIV medical care is defined as attendance at first medical appointment within 90 days of the current test date. Attendance at first medical appointment can be confirmed by client report, HIV care provider report, or HIV surveillance record check. To account for missing data on linkage to care, both minimum and maximum percentages were calculated.§ A primary goal of the National HIV/AIDS Strategy is to link 85.0% of all new positives to HIV medical care within 90 days of diagnosis (4).

HIV testing records submitted to CDC by June 2, 2014, were analyzed and stratified by 23 site types in health care and non–health care settings,¶ selected racial/ethnic categories (whites, blacks, and Hispanics), and selected target populations (MSM, heterosexual males, and heterosexual females).**

In 2013, a total of 3,343,633 CDC-funded HIV tests were conducted in the United States; HIV test setting data were available for 3,276,594 (98.0%). In health care settings, the highest percentages of tests were conducted in primary care clinics (27.2%), STD clinics (25.6%), emergency departments (15.0%), other health care settings (12.8%), and correctional facilities (10.5%). The percentage of new positives ranged from 0.2% to 0.8% and was highest in STD clinics (0.8%), unspecified outpatient facilities (0.5%), substance abuse treatment facilities (0.4%), HIV clinics (0.4%), and inpatient facilities (0.4%). In non–health care settings, the highest percentage of tests were conducted in HIV counseling and testing sites (46.7%), other non–health care settings (20.5%), and community settings (13.8%). The percentage of new positives ranged from 0.2% to 1.3% and was highest for partner services field visits (i.e., field testing of sexual partners of HIV-positive persons) (1.3%); bar, club, or adult entertainment venues (1.2%); individual residences (1.1%); and HIV counseling testing sites (1.0%) (Table 1).

No HIV testing site met the National HIV/AIDS Strategy goal of 85.0% linked to HIV medical care within 90 days of diagnosis based on the minimum percentages calculated. However, using the maximum percentages calculated, the goal was met by eight of the 12 site types among health care settings, including all except for the following: unspecified outpatient facilities (0.0%), HIV clinics (19.4%), women’s health clinics (57.1%), and TB clinics (72.2%). STD clinics identified the largest percentage of new positives (0.8%) among all health care settings and also met the National HIV/AIDS Strategy linkage goal. The linkage goal also was met by seven of 11 site types in non–health care settings, including all except for the following: community settings (67.7%), individual residences (63.6%), public areas (78.7%), and syringe exchange programs (61.5%). HIV counseling and testing sites identified the largest number of new positives linked to HIV care (2,135) (Table 1).

The percentage of new positives identified in different setting types ranged from 0.0% to 0.8% among whites, 0.2% to 2.3% among blacks, and 0.1% to 1.3% among Hispanics for both health care and non–health care settings. Within health care settings, the highest percentage of new positives among whites was found in STD clinics (0.5%) and unspecified outpatient facilities (0.4%), among blacks in STD clinics (0.8%) and substance abuse treatment facilities (0.7%), and among Hispanics in STD clinics (1.0%) and dental clinics (0.5%). In non–health care settings, the highest percentage of new positives was found in bar, club, or adult entertainment venues among blacks (2.3%), Hispanics (1.3%), and whites (0.8%); partner services field visits among blacks (2.0%) and whites (0.8%); individual residences among Hispanics (1.2%); and HIV counseling testing sites among whites (0.8%) (Table 2).

In non–health care settings, among target populations, for MSM, the percentage of new positives ranged from 0.6% in syringe exchange programs to 5.8% in partner services field visits. For heterosexual men, the percentage of new positives ranged from 0.1% in bar, club, or adult entertainment venues to 0.8% in partner services field visits. For heterosexual women, the percentage of new positives ranged from 0.1% in school or educational facilities and other non–health care settings to 0.7% in partner services field visits. Other settings with the highest percentage of new positives included other non–health care settings for MSM (3.7%), community settings for heterosexual men (0.6%), individual residences for heterosexual women (0.6%), and HIV counseling and testing sites for MSM (2.7%) and heterosexual men (0.5%) (Table 3).

**Summary**

What is already known on this topic?

CDC recommends routine screening for human immunodeficiency virus (HIV) infection in health care settings for persons aged 13–64 years in areas where prevalence is ≥0.1% and for persons at increased behavioral or clinical HIV risk in non–health care settings. Targeting HIV testing and prevention efforts toward high-risk groups in non–health care settings has been shown to be necessary to identify persons with undiagnosed HIV and link them to medical care.

What is added by this report?

In 2013, the percentage of newly identified HIV-positive persons varied widely among sites in health care settings (e.g., STD clinics [0.8%] compared with other sites [0.2%–0.5%]). In non–health care settings, HIV counseling and testing sites conducted the most HIV testing and identified the largest number of new positives (3,860), for a positivity percentage of 1.0%.

What are the implications for public health practice?

These findings highlight the importance of examining program data by settings and sites to better understand which are most effective at reaching persons with undiagnosed HIV among the most affected populations and for informing decisions about program planning and allocation of HIV testing resources.

## Discussion

STD clinics identified a higher percentage of persons with new diagnoses of HIV infection (0.8%) compared with other health care settings (0.2%–0.5%). New positives identified in non–health care settings ranged from 0.2% to 1.3% overall, 0.3% to 2.3% among blacks, 0.2% to 1.3% among Hispanics, and 0.6% to 5.8% among MSM. The findings indicate that certain site types yield higher percentages of diagnoses among persons who were previously unaware of their HIV infection. They also highlight the importance of local and national program monitoring and evaluation efforts to determine which sites are most effectively providing HIV testing, identifying new positives, and linking new positives to care. Linkage to care percentages within 90 days were low for certain site types (unspecified outpatient facilities and HIV clinics) because some persons were linked after 90 days. Additionally, data from these two site types each represent a single jurisdiction and might not reflect the national linkage percentages for these types of testing sites. These findings might enable health departments and community-based organization programs to effectively allocate HIV testing resources by testing site.

Although testing in non–health care settings identified a higher percentage of new positives, such testing often is more expensive per test than testing in health care settings and might not target all hard-to-reach populations. Conversely, health care settings, which offer more efficient methods of testing and linkage, might miss undiagnosed HIV-positive persons who do not access health care.

The findings in this report are subject to at least three limitations. First, monitoring linkage is challenging. Because of missing data, minimum and maximum percentages were calculated for linkage to care; therefore, the actual percentages lie somewhere between these two values. Second, because this report focuses only on CDC-funded HIV tests, these findings are not generalizable to the entire U.S. population. Finally, because determination of new positives was based on self-report of no prior positive test results, the number of new positives might be overestimated.

Continued efforts to target HIV testing toward high HIV prevalence areas and populations at high risk can facilitate diagnoses of new positives. For example, CDC’s Expanded HIV Testing Initiative, which targets testing toward jurisdictions with a high proportion of AIDS diagnoses among blacks, has shown a significant return on investment in HIV testing, diagnosing new positives, and averting new infections (5). Activities to reduce behavioral risk factors and improve linkage to care are critical to improve health and prevent HIV transmission to partners (6,7). Focusing HIV testing efforts on the most effective sites in both health care and non–health care settings and increasing linkage to medical care could have a large impact on identifying new positives and ensuring that they receive recommended services.

## Figures and Tables

**TABLE 1:** HIV tests, persons with newly diagnosed HIV infection, and persons linked to HIV medical care, by testing setting and site type — 61 health departments and 151 community-based organizations, United States, 2013

	HIV tests*,†		Persons with newly diagnosed HIV infection§		Persons with newly diagnosed HIV infection linked to HIV medical care within 90 days¶		
			
Testing setting/Site type	No.	(%, column)	No.	(%, row)	No.	(Minimum %)	(Maximum %)
**Health care settings**							
Primary care clinic	659,306	(27.2)	2,178	(0.3)	1,202	(55.2)	(89.2)
STD clinic	621,010	(25.6)	4,766	(0.8)	2,636	(55.3)	(85.3)
Emergency department	363,064	(15.0)	1,075	(0.3)	355	(33.0)	(85.7)
Health care, other	310,159	(12.8)	918	(0.3)	558	(60.8)	(86.8)
Correctional facility	254,719	(10.5)	841	(0.3)	319	(37.9)	(88.9)
Substance abuse treatment facility	61,386	(2.5)	230	(0.4)	118	(51.3)	(90.1)
HIV clinic	49,611	(2.0)	214	(0.4)	14	(6.5)	(19.4)
Inpatient facility	39,563	(1.6)	153	(0.4)	84	(54.9)	(94.4)
Tuberculosis clinic	36,527	(1.5)	101	(0.3)	13	(12.9)	(72.2)
Outpatient facility, unspecified	11,857	(0.5)	55	(0.5)	0	(0.0)	(0.0)
Women’s health clinic	11,807	(0.5)	30	(0.3)	4	(13.3)	(57.1)
Dental clinic	2,390	(0.1)	5	(0.2)	3	(60.0)	(100.0)
**Total**	**2,421,399**	**(100.0)**	**10,566**	**(0.4)**	**5,306**	**(50.2)**	**(85.9)**
**Non–health care settings**							
HIV counseling and testing site	399,535	(46.7)	3,860	(1.0)	2,135	(55.3)	(91.8)
Non–health care, other	174,935	(20.5)	716	(0.4)	134	(18.7)	(88.2)
Community setting	118,027	(13.8)	835	(0.7)	343	(41.1)	(67.7)
Public area	42,046	(4.9)	307	(0.7)	129	(42.0)	(78.7)
Commercial venue	31,435	(3.7)	188	(0.6)	79	(42.0)	(91.9)
School or educational facility	26,884	(3.1)	65	(0.2)	38	(58.5)	(86.4)
Field visit (partner services)	21,848	(2.6)	286	(1.3)	218	(76.2)	(96.0)
Bar, club, or adult entertainment	21,243	(2.5)	253	(1.2)	111	(43.9)	(87.4)
Shelter or transitional housing	13,051	(1.5)	55	(0.4)	27	(49.1)	(96.4)
Syringe exchange program	3,975	(0.5)	17	(0.4)	8	(47.1)	(61.5)
Individual residence	2,216	(0.3)	25	(1.1)	7	(28.0)	(63.6)
**Total**	**855,195**	**(100.0)**	**6,607**	**(0.8)**	**3,229**	**(48.9)**	**(87.6)**
Missing/Invalid	67,039	(2.0)	253	(0.4)	17	(6.7)	(53.1)
**Overall total**	**3,343,633**	**(100.0)**	**17,426**	**(0.5)**	**8,552**	**(49.1)**	**(86.5)**

**Abbreviations:** HIV = human immunodeficiency virus; STD = sexually transmitted disease.

* HIV tests are the outcome of one or more individual HIV tests performed to determine a person’s HIV status. During one testing event, a person might be tested once (e.g., one rapid test or one conventional test) or multiple times (e.g., one rapid reactive test followed by one conventional test to confirm a preliminary HIV-positive test result). Valid HIV tests are defined as records for which a test technology (conventional, rapid, nucleic acid amplification testing, or other) was reported or a test result (positive, negative, indeterminate, or invalid) was reported.

† For site types in health care settings, the denominator was the total number of HIV tests in health care settings (2,421,399). For site types in non–health care settings, the denominator was the total number of HIV tests in non–health care settings (855,195). For missing/invalid data, the denominator was the overall total number of HIV tests (3,343,633).

§ Persons who tested HIV-positive and did not report a prior positive test result were categorized as persons with newly identified HIV infection.

¶ Minimum percentages include missing data in the denominator and likely underestimate performance. Maximum percentages exclude missing data from the denominator and likely overestimate performance.

**TABLE 2:** HIV tests and persons with newly diagnosed HIV infection, by testing setting and site type and race/ethnicity — 61 health departments and 151 community-based organizations, United States, 2013

	HIV tests*						Persons with newly diagnosed HIV infection					
		
	Whites		Blacks		Hispanics		Whites		Blacks		Hispanics	
						
Testing setting/Site type	No.	(%, column)	No.	(%, column)	No.	(%, column)	No.	(%)	No.	(%)	No.	(%)
**Health care settings**												
Primary care clinic	158,589	(24.0)	280,172	(25.4)	167,387	(33.3)	509	(0.3)	1,031	(0.4)	465	(0.3)
STD clinic	178,528	(27.0)	314,687	(28.5)	93,010	(18.5)	935	(0.5)	2,624	(0.8)	926	(1.0)
Emergency department	68,838	(10.4)	181,346	(16.4)	91,333	(18.2)	144	(0.2)	693	(0.4)	198	(0.2)
Health care, other	91,557	(13.9)	132,097	(12.0)	68,757	(13.7)	126	(0.1)	542	(0.4)	207	(0.3)
Correctional facility	75,403	(11.4)	116,691	(10.6)	48,263	(9.6)	154	(0.2)	543	(0.5)	112	(0.2)
Substance abuse treatment facility	29,579	(4.5)	19,428	(1.8)	8,905	(1.8)	68	(0.2)	133	(0.7)	19	(0.2)
HIV clinic	25,212	(3.8)	20,425	(1.8)	3,026	(0.6)	76	(0.3)	125	(0.6)	8	(0.3)
Inpatient facility	9,319	(1.4)	17,025	(1.5)	9,922	(2.0)	29	(0.3)	73	(0.4)	25	(0.3)
Tuberculosis clinic	11,036	(1.7)	14,230	(1.3)	7,825	(1.6)	14	(0.1)	73	(0.5)	7	(0.1)
Outpatient facility, unspecified	7,130	(1.1)	2,380	(0.2)	1,686	(0.3)	26	(0.4)	14	(0.6)	7	(0.4)
Women’s health clinic	4,556	(0.7)	4,425	(0.4)	2,298	(0.5)	3	(0.1)	24	(0.5)	2	(0.1)
Dental clinic	473	(0.1)	1,229	(0.1)	584	(0.1)	0	(0.0)	2	(0.2)	3	(0.5)
**Total**	**660,220**	**(100.0)**	**1,104,135**	**(100.0)**	**502,996**	**(100.0)**	**2,084**	**(0.3)**	**5,877**	**(0.5)**	**1,979**	**(0.4)**
**Non–health care settings**												
HIV counseling and testing site	112,067	(50.2)	148,932	(41.3)	107,792	(52.4)	872	(0.8)	1,862	(1.3)	924	(0.9)
Non–health care, other	40,795	(18.3)	89,048	(24.7)	30,737	(14.9)	105	(0.3)	478	(0.5)	86	(0.3)
Community setting	22,426	(10.0)	64,930	(18.0)	24,646	(12.0)	98	(0.4)	581	(0.9)	130	(0.5)
Public area	7,330	(3.3)	18,897	(5.2)	11,974	(5.8)	25	(0.3)	192	(1.0)	67	(0.6)
Commercial venue	9,250	(4.1)	7,047	(2.0)	11,261	(5.5)	56	(0.6)	61	(0.9)	47	(0.4)
School or educational facility	7,867	(3.5)	12,202	(3.4)	4,778	(2.3)	10	(0.1)	41	(0.3)	11	(0.2)
Field visit (partner services)	8,017	(3.6)	8,216	(2.3)	4,103	(2.0)	63	(0.8)	164	(2.0)	43	(1.0)
Bar, club, or adult entertainment	9,333	(4.2)	3,034	(0.8)	6,953	(3.4)	77	(0.8)	70	(2.3)	89	(1.3)
Shelter or transitional housing	4,447	(2.0)	5,829	(1.6)	2,150	(1.0)	9	(0.2)	29	(0.5)	13	(0.6)
Syringe exchange program	1,595	(0.7)	1,112	(0.3)	966	(0.5)	3	(0.2)	9	(0.8)	4	(0.4)
Individual residence	312	(0.1)	1,574	(0.4)	250	(0.1)	2	(0.6)	19	(1.2)	3	(1.2)
**Total**	**223,439**	**(100.0)**	**360,821**	**(100.0)**	**205,610**	**(100.0)**	**1,320**	**(0.6)**	**3,506**	**(1.0)**	**1,417**	**(0.7)**
Missing/Invalid	18,314	(2.0)	41,060	(2.7)	4,452	(0.6)	41	(0.2)	188	(0.5)	11	(0.2)
**Overall total**	**901,973**	**(100.0)**	**1,506,016**	**(100.0)**	**713,058**	**(100.0)**	**3,445**	**(0.4)**	**9,571**	**(0.6)**	**3,407**	**(0.5)**

**Abbreviations:** HIV = human immunodeficiency virus; STD = sexually transmitted disease.

* For site types in health care settings, the denominator was the total number of HIV tests (660,220 for whites; 1,104,135 for blacks; and 502,996 for Hispanics). For site types in non–health care settings, the denominator was the total number of HIV tests (223,439 for whites; 360,821 for blacks; and 205,610 for Hispanics). For missing/invalid data, the denominator was the overall total number of HIV tests (901,973 for whites; 1,506,016 for blacks; and 713,058 for Hispanics).

**TABLE 3:** HIV tests and persons with newly diagnosed HIV infection, by target populations and non–health care test site type* — 61 health departments and 151 community-based organizations, United States, 2013

	HIV tests						Persons with newly diagnosed HIV infection					
		
	MSM		Heterosexual men		Heterosexual women		MSM		Heterosexual men		Heterosexual women	
						
Non–health care site type	No.	(%, column)	No.	(%, column)	No.	(%, column)	No.	(%)	No.	(%)	No.	(%)
HIV counseling and testing site	89,596	(59.2)	82,168	(43.6)	94,912	(38.7)	2,392	(2.7)	421	(0.5)	350	(0.4)
Non–health care, other	6,959	(4.6)	36,949	(19.6)	72,072	(29.4)	255	(3.7)	164	(0.4)	104	(0.1)
Community setting	15,040	(9.9)	33,694	(17.9)	38,405	(15.7)	297	(2.0)	197	(0.6)	141	(0.4)
Public area	7,550	(5.0)	9,459	(5.0)	10,675	(4.4)	146	(1.9)	40	(0.4)	38	(0.4)
Commercial venue	11,011	(7.3)	5,923	(3.1)	5,352	(2.2)	113	(1.0)	14	(0.2)	9	(0.2)
School or educational facility	3,064	(2.0)	5,933	(3.2)	9,725	(4.0)	39	(1.3)	2	(0.0)	11	(0.1)
Field visit (partner services)	2,192	(1.4)	7,416	(3.9)	7,721	(3.2)	127	(5.8)	59	(0.8)	57	(0.7)
Bar, club, or adult entertainment	13,600	(9.0)	1,397	(0.7)	1,401	(0.6)	225	(1.7)	1	(0.1)	4	(0.3)
Shelter or transitional housing	1,061	(0.7)	4,194	(2.2)	3,314	(1.4)	20	(1.9)	7	(0.2)	8	(0.2)
Syringe exchange program	941	(0.6)	678	(0.4)	756	(0.3)	6	(0.6)	3	(0.4)	3	(0.4)
Individual residence	302	(0.2)	441	(0.2)	711	(0.3)	7	(2.3)	1	(0.2)	4	(0.6)
**Total**	**151,316**	**(100.0)**	**188,252**	**(100.0)**	**245,044**	**(100.0)**	**3,627**	**(2.4)**	**909**	**(0.4)**	**729**	**(0.3)**

**Abbreviations:** HIV = human immunodeficiency virus; MSM = men who have sex with men.

* Data used to identify target populations are required for all tests conducted in non–health care settings but are only required for HIV-positive persons who are tested in health care settings.

## Footnotes

* HIV tests are the outcome of one or more individual HIV tests performed to determine a person’s HIV status. During one testing event, a person might be tested once (e.g., one rapid test or one conventional test) or multiple times (e.g., one rapid test followed by one conventional test to confirm a preliminary HIV-positive test result).

† Health departments in the 50 states, the District of Columbia, Puerto Rico, the U.S. Virgin Islands, and eight directly funded city/county health departments (Baltimore. Maryland; Chicago, Illinois; Fulton County, Georgia; Houston, Texas; Los Angeles County, California; New York City, New York; Philadelphia, Pennsylvania; and San Francisco, California).

§ The minimum was calculated by including missing/invalid data in the denominator, and the maximum was calculated by excluding missing/invalid data from the denominator.

¶ A health care setting is one that provides both medical diagnostic and treatment services (e.g., inpatient facilities, outpatient facilities, and emergency departments). A non–health care setting is one that does not provide both medical diagnostic and treatment services (e.g., HIV counseling and testing sites and community settings).

** Men who have sex with men are males who reported male-to-male sexual contact in the past 12 months. Heterosexual males are those who only reported heterosexual contact with a female in the past 12 months. Heterosexual females are those who only reported heterosexual contract with a male in the past 12 months. To determine target populations, CDC requires the collection of data on all tested persons in non–health care settings but only on all HIV-positive persons in health care settings.
